# The Influence of Psychological Status on Acupuncture for Postprandial Distress Syndrome: A Subgroup Analysis of a Multicenter, Randomized Controlled Trial

**DOI:** 10.1155/2022/1614648

**Published:** 2022-01-31

**Authors:** Na-Na Yang, Jing-Wen Yang, Chun-Xia Tan, Yue-jie Li, Yu Wang, Ling-Yu Qi, Cun-Zhi Liu

**Affiliations:** International Acupuncture and Moxibustion Innovation Institute, School of Acupuncture-Moxibustion and Tunia, Beijing University of Chinese Medicine, Beijing, China

## Abstract

**Background:**

Postprandial distress syndrome (PDS) is accompanied by a high incidence of mood disorder. Acupuncture is an effective method in relieving dyspepsia symptoms; however, the impact of psychological status on acupuncture for PDS remains mysterious.

**Methods:**

This secondary analysis of a multicenter, randomized controlled trial aims to evaluate the influence of anxiety and depression on acupuncture for PDS. 138 patients received the same acupuncture treatment and were followed up until week 16. The 2 primary outcomes were the response rate based on overall treatment effect and the elimination rate of all 3 cardinal symptoms after 4 weeks of treatment.

**Results:**

Of 114 patients, 31 were anxiety patients and 83 were nonanxiety patients or 32 were depressive patients and 82 were nondepressive patients. The response rate and elimination rate at week 4 were 77.4% and 9.7% in anxiety patients versus 84.3% and 27.7% in nonanxiety patients, respectively (*P* = 0.388; *P* = 0.041). No significant difference was noted in the response rate (*P* = 0.552) and elimination rate (*P* = 0.254) at week 4 between nondepressive and depressive patients. There was no significant intergroup difference in the response rate and elimination rate between non-mood-disorder and mood disorder patients (*P* > 0.05) during the 12-week post-treatment follow-up, except for the response rate at week 8 (*P* < 0.05).

**Conclusion:**

The effect of acupuncture on response rate was similar for both non-mood-disorder and mood disorder patients. However, anxiety but not depression had a negative influence on the elimination rate, especially in postprandial fullness.

## 1. Introduction

Functional dyspepsia (FD), a chronic functional gastrointestinal disorder (FGID), is characterized by upper abdominal symptoms rooting in the gastroduodenal region, without identified organic causes [1]. Two subgroups were proposed by the Roman III consensus and reiterated in the Roman IV revision: postprandial distress syndrome (PDS) with meal-related symptoms of postprandial fullness and early satiation, and epigastric pain syndrome (EPS) with meal-unrelated epigastric pain and burning [2]. PDS is the most common subtype and was reported to have the greatest negative effect on substantially impaired quality of life [3]. Despite the familiar occurrence of PDS with high prevalence and impressive medical expenses, developing treatments for FD is challenging [4].

Traditional PDS has been conceptualized as brain-gut disorders, with subgroups of patients demonstrating motility abnormalities and psychological distress [5]. Meanwhile, anxiety and depression predicted dyspepsia symptom severity independent of gastric sensorimotor function, indicating the etiological significance of anxiety and depression in FD [6]. Besides, antidepressants are frequently prescribed to treat FD [7]. Therefore, PDS was accompanied by a high incidence of mood disorder and could affect the treatment and prognosis.

Acupuncture has been sufficiently demonstrated as a potentially effective therapy in treating gastrointestinal symptoms. The efficacy of acupuncture in ameliorating the dyspepsia symptoms, including postprandial fullness and early satiation, was manifested by several randomized clinical trials [8–11]. Meanwhile, our previous trial has also shown that the effects of acupuncture were manifested after 4 weeks of treatment and were maintained during 12-week post-treatment follow-up [12]. However, these studies were focused on the treatment approach used for FD, and hence it was unclear whether psychological status affected the efficacy of acupuncture in patients with PDS. In this subgroup analysis, we sought to evaluate the influence of anxiety and depression on acupuncture for PDS.

## 2. Methods

### 2.1. Data Source, Randomization, and Blinding

Detailed descriptions of the protocol of the previous trial were published [12]. Briefly, this trial was a multicenter, randomized, sham-controlled trial conducted between April 2017 and January 2019 at 5 tertiary hospitals in China. The original trial was approved by the institutional review boards (ISRCTN registry number: ISRCTN12511434) and ethics committees of all 5 participating hospitals. Before participation, all patients provided written informed consent. A total of 278 patients were randomly assigned in a ratio of 1 : 1 to undergo acupuncture or sham acupuncture by a central web-based randomization system. Stratified block randomization was used, with a block size of 6. A randomization sequence was generated by a nonparticipating biostatistician in this trial. Patients, outcomes assessors, and statistical analysts were blinded to group assignment until the analysis was completed. Acupuncturists were not blinded to the treatment.

### 2.2. Interventions

**Figure 1:**
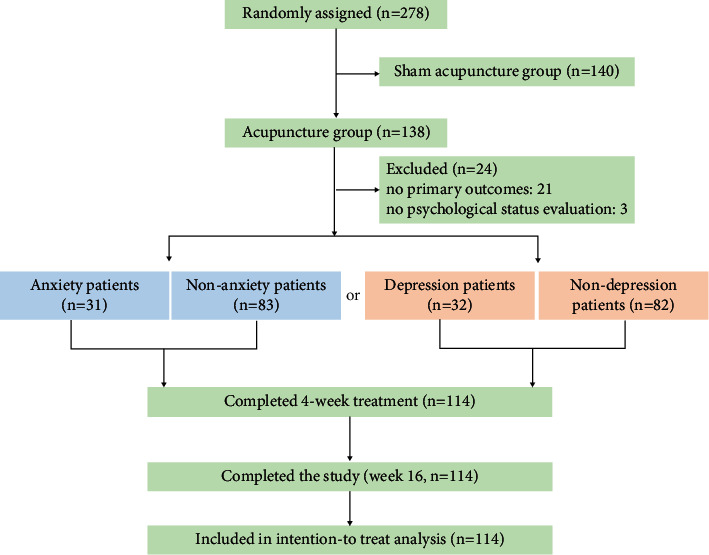
Study flow diagram.

These patients received acupuncture (138 patients) or sham acupuncture (140 patients) stimulation thrice per week with a 20-min duration over 4 weeks and were followed up until week 16 (Figure 1). In this subgroup analysis, only patients in the acupuncture group were included. Acupuncture prescription including 8 obligatory and 1 additional acupoints was manipulation by clinically experienced acupuncturists, who were twirling, rotating, lifting, and thrusting each needle for 30 seconds to achieve the de qi sensation. The obligatory acupoints were Baihui (DU20), Danzhong (RN17), Zhongwan (RN12), Qihai (RN6), and bilateral Tianshu (ST25), Neiguan (PC6), Zusanli (ST36), and Gongsun (SP4). Additional acupoints were chosen individually: Taibai (SP3) for weakness of the qi of the spleen and stomach, Taichong (LR3) for depression of the qi of the liver, and Neiting (ST44) for damp-heat in the stomach. For the current research, a total of 24 (17.4%) patients were excluded in this analysis (21 patients with no primary outcomes and 3 patients with no psychological status evaluation); and 31 anxiety patients and 83 nonanxiety patients or 32 depressive patients and 82 nondepressive patients who received acupuncture treatment were included in this subgroup analysis.

### 2.3. The Evaluation of the Psychological Status

The psychological status of the patients was evaluated via the Hospital Anxiety and Depression Scale (HADS) at the time of their inclusion into the trial. The HADS is a self-administered scale consisting of 14 items split across anxiety and depression subscales, each with a four-point ordinal response format with values ranging from 0 to 3 [13], which resulted in scale values between 0 and 21 for each scale. It was defined in two ranges for both the scales: 0–7 (nondepressive or nonanxiety patients) and 8–21 (depressive or anxiety patients) [14].

### 2.4. Outcomes

The 2 primary outcomes for this analysis were similar to the original trial [12]. Briefly, the 2 primary outcomes were the response rate based on overall treatment effect and the elimination rate of all 3 cardinal symptoms: postprandial fullness, upper abdominal bloating, and early satiation after 4 weeks of treatment. The response rate was based on the overall treatment effect questionnaire, which asked, “How were your gastric symptoms during the past week in comparison with the baseline period?” This was scored on a 7-point Likert scale, with options of “extremely improved,” “improved,” “slightly improved,” “not changed,” “slightly aggravated,” “aggravated,” or “extremely aggravated.” Patients were considered responders if they answered “extremely improved” or “improved” and were considered nonresponders otherwise. The elimination rate was defined as the proportion of patients whose score on the severity scale for all 3 cardinal symptoms (postprandial fullness, upper abdominal bloating, and early satiation) was 0 at week 4. Acupuncture was considered an effective therapy only if both primary outcomes achieved significance.

Secondary outcomes included the response rate and the elimination rate at weeks 8, 12, and 16.

### 2.5. Statistical Analysis

We summarized the baseline characteristics with the descriptive statistics for the intention-to-treat population. For baseline data, continuous variables, presented as the mean (SD), were tested by unpaired *t*-tests or nonparametric rank-sum tests. Categorical variables, shown as the number (percent), were assessed with *χ*^2^ tests.

The response rate and elimination rate were evaluated with *χ*^2^ tests at week 4, 8, 12, and 16 for primary and secondary outcomes. The correlations between the psychological assessments and scores of overall treatment effect, postprandial fullness, upper abdominal bloating, and early satiation were assessed with Spearman's correlation test.

All analyses were carried out with SPSS 23.0 software, with a 2-side significance level of less than 0.05.

## 3. Results

### 3.1. Patients' Characteristics

A total of 114 patients were eligible for this analysis. Demographic characteristics including age, sex, marital status, occupation, education, body mass index, disease duration, endoscopy findings, and *Helicobacter pylori* status between anxiety and nonanxiety patients or depressive and nondepressive patients were comparable, with no significant difference in parameters (Table 1).

### 3.2. The Influence of Anxiety on Acupuncture for PDS

There was no significant difference between the two groups in the response rate at week 4 (anxiety group 77.4% vs. nonanxiety group 84.3%, difference: 1.6 percentage points [95% CI, 0.6 to 4.4 percentage points], *P* = 0.388). However, the elimination rate was significantly higher in the nonanxiety patients (27.7%) than anxiety patients (9.7%) after acupuncture treatment (difference: 3.6 percentage points [95% CI, 1.0 to 12.9 percentage points], *P* = 0.041) (Table 2).

**Figure 2:**
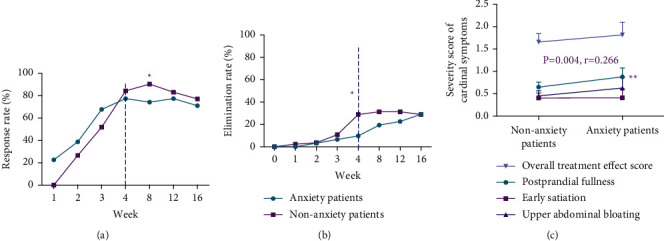
Response rate based on overall treatment effect (a) and elimination rate of all 3 cardinal symptoms (b) at each assessment time point. Spearman's related analysis between the comorbid anxiety and the score of overall treatment effect or all 3 cardinal symptoms at week 4 (c). ^*∗*^*P* < 0.05; ^*∗∗*^*P* < 0.01.

The response rate and elimination rate were gradually increased from week 1 in both groups (Figures 2(a) and 2(b)). For the response rate, no significant difference was found until week 8 (anxiety group 74.2% VS nonanxiety group 90.4%, difference: 3.3 percentage points [95% CI, 1.1 to 9.7 percentage points], *P* = 0.027). Compared with anxiety patients, nonanxiety patients have a higher response rate at week 12 and week 16; however, these between-group differences were not statistically significant (week 12: 77.4% vs. 83.1%, difference: 1.4 percentage points [95% CI, 0.5 to 4.0 percentage points], *P* = 0.484; week 16: 71.0% vs. 77.1%, difference: 1.4 percentage points [95% CI, 0.5 to 3.5 percentage points], *P* = 0.498) (Table 3). Similarly, no significant difference was noted in the elimination rate at week 8 (anxiety group 19.4% vs. nonanxiety group 31.3%, difference: 1.9 percentage points [95% CI, 0.7 to 5.2 percentage points], *P* = 0.206), week 12 (anxiety group 22.6% vs. nonanxiety group 28.9%, difference: 1.6 percentage points [95% CI, 0.6 to 4.1 percentage points], *P* = 0.360), and week 16 (anxiety group 29% vs. nonanxiety group 28.9%, difference: 1.0 percentage points [95% CI, 0.4 to 2.5 percentage points], *P* = 0.990) (Table 3).

Furthermore, we also analyzed the relationship between the comorbid anxiety and the score of overall treatment effect or all 3 cardinal symptoms using Spearman's correlation after 4 weeks of treatment. The score of anxiety was positively correlated with the severity score of postprandial fullness (*R* = 0.27, *P* = 0.004). However, the correlation analysis revealed no significant relationship between anxiety and overall treatment effect (*R* = 0.18, *P* = 0.058), upper abdominal bloating (*R* = 0.15, *P* = 0.110), or early satiation (*R* = 0.16, *P* = 0.083) (Figure 2(c)).

### 3.3. The Influence of Depression on Acupuncture for PDS

With respect to the primary outcomes, the response rate and elimination rate in depressive patients and nondepressive patients were 78.1%, 15.6% and 82.8%, 25.6%, respectively, at week 4. However, these between-group differences were not statistically significant (response rate, difference: 1.4 percentage points [95% CI, 0.5 to 3.8 percentage points], *P* = 0.552; elimination rate, difference: 1.9 percentage points [95% CI, 0.6 to 5.4 percentage points], *P* = 0.254) (Table 4).

**Figure 3:**
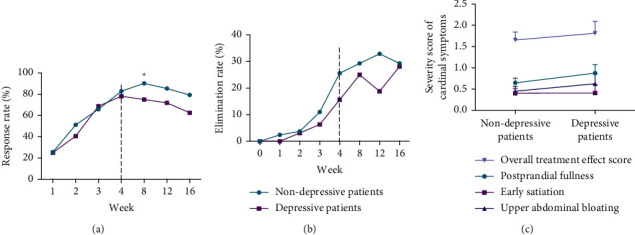
Response rate based on overall treatment effect (a) and elimination rate of all 3 cardinal symptoms (b) at each assessment time point. Spearman's related analysis between the comorbid depression and the score of overall treatment effect or all 3 cardinal symptoms at week 4 (c). ^*∗*^*P* < 0.05.

At each assessment time point during follow-up, the nondepressive patients had better results than depressive patients in the response rate and elimination rate (Figures 3(a) and 3(b)); however, it was not a significant difference except for the response rate at week 8 (depressive group 75.0% VS nondepressive group 90.2%, difference: 3.1 percentage points [95% CI, 1.0 to 9.1 percentage points], *P* = 0.035) (Table 5).

The correlation analysis revealed no significant relationship between depression and overall treatment effect (*R* = 0.17, *P* = 0.077), the postprandial fullness (*R* = 0.18, *P* = 0.053), early satiation (*R* = 0.10, *P* = 0.293), or upper abdominal bloating (*R* = 0.14, *P* = 0.152) (Figure 3(c)).

## 4. Discussion

To our knowledge, it is the first time to evaluate the impact of psychological status on the outcomes of acupuncture for PDS patients. This trial found that the beneficial effects of acupuncture in the response rate based on overall treatment effect were similar for both non-mood-disorder and mood disorder patients. However, anxiety but not depression had a negative influence on the elimination rate of all 3 cardinal symptoms after acupuncture treatment at week 4.

The putative bio-psycho-social pathophysiological model for FD underscores the importance of psychological distress in the pathogenesis of PDS. A wealth of clinical studies indicated a high degree of comorbidity between PDS and anxiety [15, 16], and anxiety comorbidity correlated with the severity of PDS symptoms [17]. A trial of the Swedish population found that 10 years later the risk of FD developing was a nearly 8-fold increase in those with higher anxiety levels and the risk of FD in those with anxiety was limited to PDS and not EPS [18]. Furthermore, anxiety status might influence the patients' attitude towards disease treatment and the efficacy. In this subgroup analysis, the elimination rate was 18.0 percentage points higher in the nonanxiety patients than anxiety patients after 4 weeks of treatment, which indicated anxiety harmed acupuncture to improve the symptoms of PDS patients. Meanwhile, the elimination rate was 11.9 and 6.3 percentage points higher in the nonanxiety patients than anxiety patients at week 8 and week 12, respectively, but there was no significant intergroup difference. Additionally, even though the results indicated that the positive association between anxiety and the severity of postprandial fullness in acupuncture treatment was possible, the correlation was not sufficiently strong. Therefore, a prospective randomized controlled trial or predesign subgroup analysis based on anxiety may be indispensable to explore the definite causal relationship between anxiety and postprandial fullness in acupuncture treatment. This was consistent with the result of a study in which increasing levels of anxiety were associated with fullness and bloating [19]. These results supported the view that anxiety had a negative influence on the elimination rate, especially in postprandial fullness.

PDS is a symptom-based systemic disease, and the response rate is based on the overall treatment effect, which suggested improvement of the response rate is very meaningful in a clinical study for PDS, although it is a subjective global evaluation. In this secondary analysis, we also noted that nonanxiety patients had 6.9, 16.2, 5.7, and 6.1 percentage point improvement over anxiety patients at week 4, week 8, week 12, and week 16, respectively. Interestingly, the response rate in anxiety subjects was higher than that in nonanxiety subjects from week 1 to week 3. A population-based study found that anxiety but not depression was an independent factor in determining health-care-seeking behavior in Chinese patients with dyspepsia [20]. Therefore, compared with nonanxiety patients, anxiety patients who were willing to receive treatment may get the higher subjective index of response rate during 4-week treatment of acupuncture, but the elimination rate was similar in these two groups.

Depression, the most common mental disorder, leads to a variety of functional diseases and may have the potential to affect the attitude of treatment in patients with diseases, thus reducing the quality of life. Our analysis revealed that the beneficial effect of acupuncture in PDS was similar for both depressive patients and nondepressive patients in the response rate and elimination rate, as assessed over treatment and follow-up. A trial with 703 community subjects provided powerful evidence to support the viewpoint that depression did not affect the risk of FD developing [18]. Besides, the correlation analysis revealed no significant relationship between depression and symptoms of PDS. Similarly, another trial with 193 patients also found that depression was not associated with fullness but had a positive association with abdominal pain, which is the main symptom of EPS. Although antidepressants including tricyclic antidepressants and selective serotonin reuptake inhibitors have been used for irritable bowel syndrome [21], their efficacy in FD management is very uncertain. A multicenter, randomized, placebo-controlled trial with 292 subjects found that antidepressants appeared to benefit some patients with ulcer-like (painful) FD, not dysmotility-like FD, and patients with delayed gastric emptying do not respond to these drugs [7]. These results indicated that the depressive level may have no effect on the improvement of symptoms in patients with PDS by acupuncture treatment.

Notably, the response rate was 16.2 or 15.2 percentage points higher in the nonanxiety or nondepressive patients than anxiety or depressive patients at week 8, with a significant difference. Studies reported an action delay between the initiation of treatment and significant clinical responses in patients with mood disorder because it needed time to reach an effective target concentration [22, 23]. Surveys such as that conducted by Tu Y have shown that acupuncture was efficacious for the severity of depressive disorder after 8-week treatment [24]. In our previous trial, there was a statistical trend for a greater reduction in the HADS score following acupuncture versus sham acupuncture at week 8, though no significant effects were found (*P* = 0.051) [12]. Therefore, it is a plausible assumption that acupuncture has the potential to improve the clinical symptoms of mood disorder patients at week 8, and then these patients were more objectively and calmly evaluating the response rate at this time.

This secondary analysis has limitations. First, this study was conducted as an unplanned, retrospective subgroup analysis, with the number of anxiety or depressive patients being smaller than that in the control group, so it may not be possible to extrapolate the findings. Second, the psychological status of the PDS patients was measured by the HADS in this study, but this scale may not provide good separation between symptoms of anxiety and depression due to presence of a strong general factor [13]. Third, although we found that mood disorder at baseline had influences on the therapeutic effect of acupuncture in PDS patients, the casual relationship between psychological status and symptoms improvement after treatment was still unclear. In our trials, acupuncture improved the symptoms of PDS at week 4 and had a reduction trend of the HADS score at week 8, and these findings indicated that the efficacy of acupuncture for psychological status may be mediated by the improvement of PDS. However, this assumption remains to be further assessed in large and well-designed clinical trials.

## 5. Conclusion

In conclusion, we present, to the best of our knowledge, the first evidence that the beneficial effect of acupuncture in the response rate was similar for both non-mood-disorder and mood disorder patients. However, anxiety but not depression had a negative influence on the elimination rate, especially in postprandial fullness, after acupuncture treatment at week 4.

## Figures and Tables

**Table 1:** Participant baseline characteristics.

Characteristic	Anxiety (*n* = 114)		Depression (*n* = 114)	
	Anxiety patients, *n* = 31	Nonanxiety patients, *n* = 83	Depressive patients, *n* = 32	Nondepressive patients, *n* = 82
Mean age (SD), *y*	42.3 (2.4)	41.0 (1.4)	41.9 (2.1)	41.1 (1.5)

Sex, *n* (%)				
Female	21 (67.7)	63 (75.9)	23 (71.9)	61 (74.4)
Male	10 (32.3)	20 (24.1)	9 (28.1)	21 (25.6)

Marital status, *n* (%)				
Married	24 (77.4)	60 (72.3)	27 (84.4)	57 (69.5)
Single	7 (22.6)	23 (27.7)	5 (15.6)	25 (30.5)

Occupation, *n* (%)				
Mental work	27 (87.1)	72 (86.7)	31 (96.9)	68 (82.9)
Manual work	4 (12.9)	11 (13.3)	1 (3.1)	14 (17.1)
Education (SD), *y*	14.7 (0.7)	15.1 (0.3)	15.2 (0.6)	14.9 (0.3)
Mean body mass index (SD), kg/m^2^	21.5 (0.7)	22.1 (0.4)	22.0 (0.7)	21.9 (0.4)
Mean disease duration (SD), *mo*	66.2 (12.2)	52.2 (5.6)	62.8 (11.0)	53.3 (6.0)

Endoscopy findings, *n* (%)^⨗^				
Normal	0 (0)	6 (11.8)	4 (19.0)	2 (3.9)
Chronic superficial gastritis	14 (66.7)	32 (62.7)	12 (57.1)	34 (66.7)
Chronic nonatrophic gastritis	7 (33.3)	13 (25.5)	5 (23.8)	15 (29.4)

*Helicobacter pylori* status, *n* (%)^℥^				
Positive	3 (10.3)	20 (26.7)	6 (20.7)	18 (24.0)
Negative	26 (89.7)	55 (73.3)	23 (79.3)	57 (76.0)

^*∗*^There was significant difference (*P* < 0.05) between the study groups. ^⨗^Data were available for 77 patients. ^℥^Data were available for 104 patients.

**Table 2:** Primary outcomes in the anxiety and nonanxiety patients.

Outcome, *n* (%)	Anxiety patients, *n* = 31	Nonanxiety patients, *n* = 83	Difference (95% CI)	*P* value^∔^
Response rate	24 (77.4)	70 (84.3)	1.6 (0.6 to 4.4)	0.388
Elimination rate	3 (9.7)	23 (27.7)	3.6 (1.0 to 12.9)	0.041^*∗*^

^∔^Calculated using the *χ*^2^ test. ^*∗*^*P* < 0.05.

**Table 3:** Secondary outcomes in the anxiety and nonanxiety patients.

Outcome, *n* (%)	Anxiety patients, *n* = 31	Nonanxiety patients, *n* = 83	Difference (95% CI)	*P* value^∔^
Response rate				
Week 8	23 (74.2)	75 (90.4)	3.3 (1.1 to 9.7)	0.027^*∗*^
Week 12	24 (77.4)	69 (83.1)	1.4 (0.5 to 4.0)	0.484
Week 16	22 (71.0)	64 (77.1)	1.4 (0.5 to 3.5)	0.498

Elimination rate				
Week 8	6 (19.4)	26 (31.3)	1.9 (0.7 to 5.2)	0.206
Week 12	7 (22.6)	24 (28.9)	1.6 (0.6 to 4.1)	0.360
Week 16	9 (29.0)	24 (28.9)	1.0 (0.4 to 2.5)	0.990

^∔^Calculated using the *χ*^2^ test. ^*∗*^*P* < 0.05.

**Table 4:** Primary outcomes in the depressive and nondepressive patients.

Outcome, *n* (%)	Depressive patients, *n* = 32	Nondepressive patients, *n* = 82	Difference (95% CI)	*P* value
Response rate	25 (78.1)	68 (82.8)	1.4 (0.5 to 3.8)	0.552
Elimination rate	5 (15.6)	21 (25.6)	1.9 (0.6 to 5.4)	0.254

**Table 5:** Secondary outcomes in the depressive and nondepressive patients.

Outcome, *n* (%)	Depressive patients, *n* = 32	Nondepressive patients, *n* = 82	Difference (95% CI)	*P* value^∔^
Response rate				
Week 8	24 (75.0)	74 (90.2)	3.1 (1.0 to 9.1)	0.035^*∗*^
Week 12	23 (71.9)	70 (85.4)	2.2 (0.9 to 6.1)	0.095
Week 16	20 (62.5)	65 (79.3)	2.3 (0.9 to 5.6)	0.065

Elimination rate				
Week 8	8 (25.0)	24 (29.3)	1.2 (0.4 to 3.1)	0.649
Week 12	6 (18.8)	27 (32.9)	2.1 (0.8 to 5.8)	0.134
Week 16	9 (28.1)	24 (29.3)	1.1 (0.4 to 2.6)	0.904

^∔^Calculated using the *χ*^2^ test. ^*∗*^*P* < 0.05.

## Data Availability

The date used to support the findings of this study are included within this article.
